# Effect of proinflammatory diet before pregnancy on gestational age and birthweight: The Japan Environment and Children's Study

**DOI:** 10.1111/mcn.12899

**Published:** 2019-11-20

**Authors:** Makiho Ishibashi, Hyo Kyozuka, Akiko Yamaguchi, Keiya Fujimori, Mitsuaki Hosoya, Seiji Yasumura, Kuse Masahito, Akiko Sato, Yuka Ogata, Koichi Hashimoto, and the Japan Environment and Children's Study (JECS) Group

**Affiliations:** ^1^ Fukushima Regional Center for the Japan Environmental and Children's Study Fukushima Japan; ^2^ Department of Obstetrics and Gynecology Fukushima Medical University School of Medicine Fukushima Japan; ^3^ Department of Pediatrics Fukushima Medical University School of Medicine Fukushima Japan; ^4^ Department of Public Health Fukushima Medical University School of Medicine Fukushima Japan

**Keywords:** dietary inflammatory index, fetal growth, food frequency questionnaires, maternal nutrition, obstetrician, preterm birth, prospective study

## Abstract

The daily diet plays a role in systematic inflammation and may be one of the causes of preterm birth. We aimed to examine the effect of a daily proinflammatory diet before pregnancy on gestational age and birthweight using a large birth cohort in Japan. We used data of singleton pregnancies in the Japan Environment and Children's Study involving live birth from 2011 to 2014 to calculate the dietary inflammatory index. We used individual meals with 30 food parameters from a semiquantitative food frequency questionnaire, which assessed diet intake before pregnancy. Participants were categorized according to the quartile of dietary inflammatory index. A multiple logistic regression model was used to estimate the risk of a proinflammatory diet on preterm birth (PTB) before 37 or 34 weeks and low birthweight (LBW) less than 2,500 or 1,500 g, accounting for maternal age, body mass index before pregnancy, smoking status, education, and household income. After applying our inclusion criteria, 89,329 participants were eligible for the present study. Multiple regression analysis showed that the proinflammatory diet had an increased risk of PTB < 34 weeks (adjusted odds ratio: 1.29, 95% confidence interval [1.07, 1.55]) and <2,500‐g LBW (adjusted odds ratio: 1.08, 95% confidence interval [1.01, 1.16]) compared with the control. In conclusion, a proinflammatory diet before pregnancy was a risk factor for PTB < 34 weeks and LBW < 2,500 g. Therefore, proinflammatory diet needs to be controlled to improve perinatal prognosis.

**Key messages**

- Preterm birth, mainly caused by intrauterine infection, is related to neonatal mortality and morbidity.
- In addition to bacterial infections from the vagina, an inflammatory diet can also cause intrauterine inflammation, causing preterm birth.
- Our study found that an inflammatory diet before pregnancy was related to preterm birth, low birthweight infants, and hypertensive disorder of pregnancy.

**Abbreviations**
aOR: adjusted odds ratio
BMI: body mass index
CI: confidence interval
DII: dietary inflammatory index
FFQs: food frequency questionnaires
HDP: hypertensive disorder of pregnancy
HPC: hydroxyprogesterone
JECS: Japan Environment and Children's Study
LBW: low birthweight infants
PTB: preterm birth
*SD*: standard deviation
SGA: small for gestational age

## 1 INTRODUCTION

Preterm birth (PTB), which can result in low birthweight (LBW) infants, is a major cause of neonatal morbidity and mortality. As preterm infants have immature fetal growth, complications such as cerebral haemorrhage and respiratory distress syndrome can occur (Altman, Vanpée, Cnattingius, & Norman, 2011). In Japan, PTB is a public concern because there was an increase in the rate of PTB before 37 weeks (4.5% to 5.6%), LBW < 2,500 g (6.5% to 9.5%), and <1,500 g (0.53% to 0.75%) from 1990 through 2015 (Japanese Ministry of Health, Labor and Welfare, 2015). Because the randomized control study by Meis et al. (2003), the effectiveness of intramuscular 17‐hydroxyprogesterone caproate for preventing PTB has been widely recognized worldwide. Despite the widely recognized effectiveness of 17‐hydroxyprogesterone caproate on recurrent spontaneous PTB, this drug also has potential problems, including lack of effectiveness, for patients without any history of previous spontaneous PTB and pricing concerns (Nelson et al., 2017). Therefore, other measures to reduce the risk of PTB that can be applied to a wide range of pregnant women are required.

One of the main causes of PTB is local inflammation in the uterus (Kyozuka et al., 2017). Intrauterine inflammation is thought to be mainly due to bacterial ascending infection from the vagina, resulting in spontaneous PTB (Romero & Mazor, 1988). A recent study indicated that maternal infection is not required for PTB. Maternal systematic inflammation induced by daily diet is thought to be another cause of PTB. A previous randomized control study of Norwegian women showed that intervention during the second trimester by controlling diet or antiinflammatory diet reduced the risk of PTB (Khoury, Henriksen, Christophersen, & Tonstad, 2005). Scholl, Chen, Goldberg, Khusai, and Stein (2011) reported that higher levels of sensitive C‐reactive protein (CRP), which is a systematic biomarker induced by inflammatory diets, were associated with the risk of preterm delivery.

In nonpregnant adult, diet is thought to play an important role in the regulation of chronic inflammation. For example, a high‐calorie, high‐fat diet, such as diets including Western food, promotes inflammation and consumption of Western food exposes the body to repeated inflammation (Lopez‐Garcia et al., 2004). As a result, several diseases such as cardiovascular disease, diabetes mellitus, thrombosis, asthma, and depression may occur (Giugliano, Ceriello, & Esposito, 2006; Pearson et al., 2003; Ramallal, Toledo, Martínez‐González, & Hernández‐Hernández, 2015). On the other hand, there are many vegetables and foods rich in minerals, such as traditional Japanese food and Mediterranean food that have a lower inflammatory effect (Guo et al., 2012; Tada, 2011). In recent years, the concept of proinflammatory and antiinflammatory diets has been reported. The dietary inflammatory index (DII) is a method to assess the inflammatory potential of an individual's diet (Shivappa, Steck, Hurley, Hussey, & Hébert, 2014). The DII has been proved to associate with well‐known inflammation related condition such as obesity (Ruiz‐Canela et al., 2015), asthma (Wood, Shivappa, Berthon, Gibson, & Hebert, 2015), and colorectal cancer (Jayedi, Emadi, & Shab‐Bidar, 2018).

Although numerous studies have examined the relation between daily diet and occurrence of several diseases, few large birth cohort studies have been conducted with regard to the correlation between the proinflammatory/antiinflammatory contents of daily diets before pregnancy and obstetrical complications.

Hence, we investigated the effect of a proinflammatory diet before pregnancy by mean of DII score on gestational age and birthweight in the largest Japanese birth cohort study.

## 2 MATERIALS AND METHODS

### 2.1 Study design

In this study, data from the Japan Environmental Children's Study (JECS), a government‐funded birth cohort study started in January 2011, were used. This survey investigated the effect of several environmental factors on children's health (Kawamoto et al., 2014). Eligibility requirements of JECS participants (mothers) were as follows: (a) living in the study area at the time of application and were expected to live in Japan in the near future; (b) expected delivery date between August 1, 2011, and mid‐2014; and (c) could participate without difficulty (i.e., they could answer the self‐management questionnaire). The target recruitment rate was >50% of all eligible mothers. Written informed consent was obtained from all participating women.

The JECS protocol was reviewed and approved by the Ministry of the Environment's Institutional Review Board on Epidemiological Studies and by the Ethics Committees of all participating institutions. The JECS was conducted in accordance with the Helsinki Declaration and other nationally valid regulations and guidelines.

## 3 DATA COLLECTION

We used the dataset released in June 2016 (dataset: jecs‐ag‐20160424) for this study. This data set consisted of 4 types of information: (1) Self‐reported questionnaire obtained around the 1^st^ trimester, including the medical background, or food frequency questionnaires (FFQs); (2) Self‐reported questionnaire collected during their second/third trimester, including socioeconomic status such as maternal education or household income: (3) Obstetrics outcome which was retrieved from medical records of each subject's institution. (4) Maternal blood sample collected during their first trimester. The FFQ was completed during the first trimester, and diet intake was assessed before pregnancy. This tool, which was used in the JECS, has been validated as a self‐administrated diet questionnaire in previous Japanese epidemiological studies (Yokoyama et al., 2016).

In the present study, we excluded cases with insufficient data, multiple pregnancies, or delivery before 22 weeks.

## 4 CALCULATION OF DII

The DII score is a comprehensive indicator of daily inflammatory and antiinflammatory meal contents developed by Shivappa et al. (Shivappa, Steck, Hurley, Hussey, & Hébert, 2014). The greater the DII score, the more proinflammatory diet. A more negative value indicates a more antiinflammatory diet.

In the present study, the 30 food parameters, including energy, carbohydrate, protein, total fat, alcohol, fibre, cholesterol, saturated fat, monounsaturated fatty acids (MUFAs), polyunsaturated fatty acids (PUFAs), fatty acids (n–3 and n–6 FAs), niacin, thiamin, riboflavin, iron, magnesium, zinc, selenium, vitamin A, B‐12, B‐6, C, D, E, folic acid, β‐carotene, garlic, ginger, and onion were obtained from each participants' FFQ. The DII score of each participant was calculated according to Shivappa et al. (2014a). First, the dietary data were linked to a worldwide database that provided a robust estimate of the mean and standard deviation (*SD*) for each parameter included in the DII (Shivappa, Steck, Hurley, Hussey, & Hébert, 2014). The Z score was calculated by subtracting the standard global mean from the reported amount and dividing the result by the *SD*. The Z scores were not normally distributed (right skewing); thus, the Z score of each value was converted to a centred percentile score. Then, the centred percentile score for each food parameter was multiplied by the respective food parameter effect score (obtained by reviewing a total of 1943 research articles to determine the relationship between the food parameters and inflammation, as well as by scoring) to obtain a food parameter‐specific DII score, which were all summed to create the overall DII score for each participant. *DII* = *I*_1_·*P*_1_ + *I*_2_·*P*_2_ + … + *I*_30_·*P*_30_, where *I* is the food parameter effect score considering the effect of inflammation obtained from reviewed research articles, and *P* is the food specific centred percentile score derived from food data. The DII minimum/maximum in nonpregnant populations is reported to range from −8.87 to +7.98 (Shivappa, Steck, Hurley, Hussey, & Hébert, 2014). DII score has already been validated in nonpregnant adults to correlate with various inflammatory markers including CRP, tumour necrosis factor alpha (TNF‐α), and interleukin (IL)‐6 (Shivappa et al., 2014; Shivappa et al., 2017).

## 5 OBSTETRICS OUTCOMES AND CONFOUNDING FACTORS

PTB was classified into two categories: delivery before 37 weeks and before 34 weeks. LBW was also categorized into two categories: <2,500 and <1,500 g. Fetal growth restriction was evaluated using small for gestational age (SGA). SGA was defined as a birthweight below −1.5 standard deviation (*SD*) corrected for gestational age and sex according to Itabashi, Miura, Uehara, and Nakayama (2014). In this study, hypertensive disorder of pregnancy (HDP) was defined the new onset of hypertension (≥140/90 mmHg) after the 20th gestational week in a previously normotensive woman. Leukocytosis was defined as a white blood cell (WBC) count of >12,000 in the maternal blood sample. Confounding factors for this study were determined by clinical importance, that is, those believed to be related to PTB and dietary habits. The following items were used as confounding factors: maternal age, maternal BMI before pregnancy, maternal smoking status, education state of mother, and annual household income. Maternal age was categorized into six age groups: ≤19, 20–24, 25–29, 30–34, 35–39, and ≥40 years. Maternal body mass index (BMI) before pregnancy was calculated by dividing the height (m) by the square of the body weight (kg) using the height and weight. We categorized participants into three BMI groups as follows: <18.5, 18.5–25.0, and ≥25.0 kg/m^2^. T1 data provided information on their smoking status during first trimester: “never smoked,” “quit smoking before pregnancy,” “quit smoking during early pregnancy,” and “kept smoking during pregnancy.” “Kept smoking during pregnancy” was defined as the smoking category; otherwise, it was defined as nonsmoking. Maternal education was categorized into four groups (junior high school: <10, high school: 10–12 years, professional school or university: 13–16 years, and graduate school: ≥17 years). Annual household income was categorized into four levels (<2,000,000; 2,000,000–5,999,999; 6,000,000–9,999,999; and ≥10,000,000 JPY; Kyozuka et al., 2019).

## 6 STATISTICAL ANALYSES

The participants were categorized according to quartiles (Q1 was for the most antiinflammatory group and Q4 for the most proinflammatory group). Maternal characteristics were summarized according to each group. One‐way analysis of variance and the chi‐square test was used to compare the continuous and categorical variables, respectively. Adjusted odds ratios (aORs) and 95% confidence intervals (CIs) for PTB, LBW, SGA, and HDP were calculated using a multiple logistic regression model, accounting for maternal age, maternal BMI before pregnancy, maternal education, maternal smoking status, and household income. We accomplished this by using dummy variables for categorical variables composed of more than three categories. SPSS version 21 (IBM Corp., Armonk, NY) was used for the statistical analyses. A *P* value <.05 indicated statistical significance.

## 7 RESULTS

**Figure 1:**
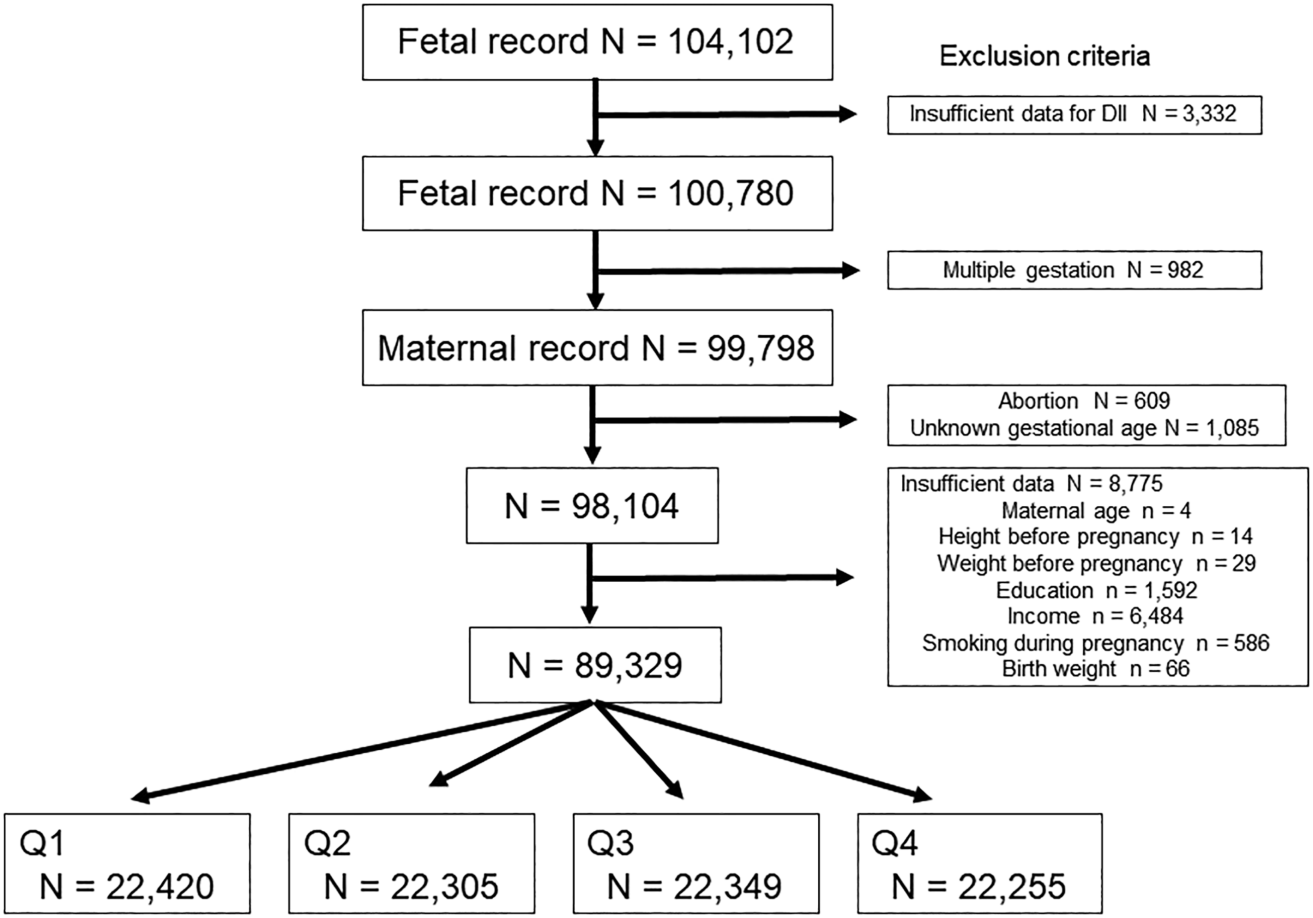
Study enrollment flowchart. Abbreviations: BMI, body mass index; DII, diet‐derived inflammation index

**Figure 2:**
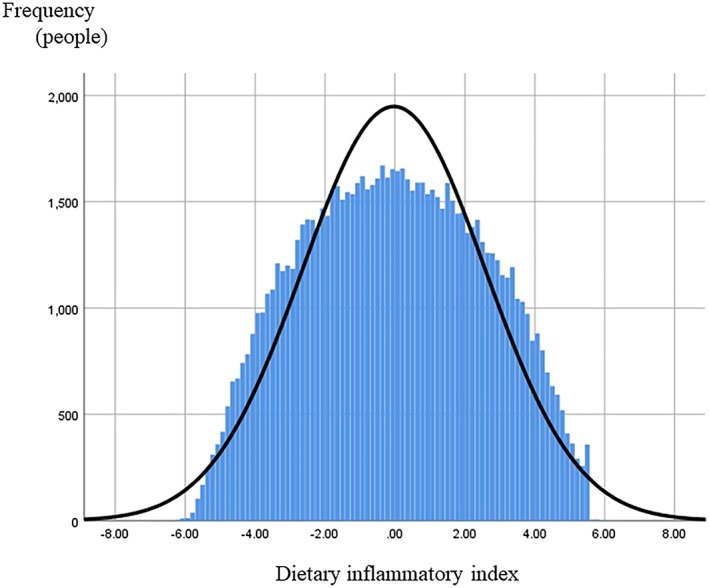
The frequency distribution of DII score. The horizontal axis indicates DII score and the vertical axis indicates the number of participants. DII score ranged from −6.16 to 5.80. The curved line indicates the normal distribution curve. Abbreviation: DII, diet‐derived inflammation index

The total number of fetal records from infants delivered between 2011 to 2014 in the JECS was 104,102. Of these, 3,332 and 982 infants were excluded due to insufficient data for DII and multiple gestation, respectively. Then, 609, 1,085, and 8,775 participants were excluded for the reasons of abortion, unknown gestational age, and insufficient data, respectively. After applying our inclusion criteria, 89,329 participants were eligible for the present study and categorized into four groups according to quartiles (Figure 1). Figure 2 shows the frequency distribution of the DII score. The DII score ranged from −6.16 to +5.80.

## 8 MATERNAL MEDICAL AND SOCIOECONOMIC BACKGROUND AND OBSTETRIC OUTCOMES

Table 1 summarizes the maternal medical background and obstetric outcomes according to quartiles for DII score. There was a significant difference in age, BMI before pregnancy, smoking during pregnancy, maternal education, and household income among the four groups (*P* < .01). Whereas maternal age <20 years was the most common in the highest DII score quartile (the most proinflammatory group), maternal age >40 years was the most common in the lowest DII score quartile (the most proinflammatory group). The proportion of low maternal education (<10 years) and low household income (<2,000,000 JPY) increased with increasing quartiles of DII. The ratio of leukocytosis, which was defined as WBC count over 12,000 during the first trimester, also increased with each increasing quartile of DII.

**Table 1:** Maternal medical background and obstetric outcomes

	Quartile for DII				
Variable	Q1 (*n* = 22,420)	Q2 (*n* = 22,350)	Q3 (*n* = 22,349)	Q4 (*n* = 22,255)	*P* value
	Most antiinflammatory			Most proinflammatory	
Maternal medical background					
DII before pregnancy	−3.41 (0.90)	−1.04 (0.58)	0.96 (0.59)	3.37 (0.91)	<.01^a^
Maternal age, years mean (*SD*)	32.1 (4.8)	31.7 (4.9)	31.1 (4.9)	29.8 (5.3)	<.01^a^
Maternal age category (years), %					
<20	0.3	0.5	0.5	1.2	
20–24	4.9	6.2	8.1	13.7	
25–29	23.8	25.6	28.5	32.7	<.01^b^
30–34	38.0	37.4	36.5	32.0	
35–39	27.2	25.2	22.1	17.2	
>40	5.8	5.2	4.3	3.2	
BMI before pregnancy (kg/m^2^), %					
<18.5	14.9	15.9	16.3	16.8	
18.5 to 25.0	74.4	74.2	73.6	71.7	<.01^b^
>25	10.7	9.9	10.2	11.5	
Smoked during pregnancy, %	3.7	3.8	4.5	6.4	<.01^b^
Maternal education (years), %					
<10	3.3	3.5	4.4	6.8	
10–12	25.9	27.7	31.1	38.0	
13–16	44.8	43.5	42.3	38.8	<.01^b^
≥17	26.0	25.3	22.2	16.4	
Household income, (JPY), %					
<2,000,000	5.0	4.5	5.2	7.8	
2,000,000–5,999,999	65.6	66.6	67.9	70.4	
6,000,000–9,999,999	24.0	24.2	22.8	18.8	<.01^b^
≥10,000,000	5.3	4.7	4.1	3.0	
Gestational week at blood collection, (weeks) mean (*SD*)	11.14 (1.82)	11.10 (1.81)	11.09 (1.83)	11.12 (1.83)	<.01^a^
White blood cells (counts per litre) mean (*SD*)^c^	8,009 (1927)	8,016 (1935)	8,057 (1937)	8,076 (1936)	<.01^a^
White blood cells > 12,000 (counts per litre), %	2.6	2.5	2.9	3.0	.02^b^
Obstetric outcome					
PTB < 37 weeks, %	5.0	4.9	5.2	5.0	.65^b^
PTB < 34 weeks, %	1.0	1.0	1.0	1.2	.13^b^
LBW < 2,500 g, %	8.3	8.2	8.7	8.9	.03^b^
LBW < 1,500 g, %	0.6	0.5	0.6	0.6	.38^b^
SGA, %	5.2	4.8	4.9	5.2	.10^b^
HDP, %	2.7	2.8	2.8	3.1	.04^b^

Abbreviations: BMI, body mass index; DII, dietary inflammatory index; HDP, hypertensive disorder of pregnancy; JPY, Japanese Yen; LBW, low birthweight; PTB, preterm birth; *SD*, standard deviation; SGA, small for gestational age.

a *P* value, one‐way analysis of variance.

b *P* value, chi‐square test.

c White blood cell count consists of the numbers of 19,952, 199,78, 20,116, and 20,156 for Q1, Q2, Q3 and Q4, respectively.

With regard to obstetrics outcome, significant differences in the occurrence of LBW < 2,500 g and HDP were seen (*P* = .03 and *P* = .04, respectively). These occurrences increased with the increase in DII category.

## 9 DII AND RISK OF OBSTETRIC COMPLICATION

Table 2 summarizes the association between DII category and risk of obstetric complications (PTB, LBW, SGA, and HDP). When we considered Q1 (the most antiinflammatory groups) as reference, multiple logistic regression showed that Q4 (the most proinflammatory group) had an increased risk of PTB < 34 weeks (aOR: 1.37, 95% CI [1.08, 1.73]), LBW < 2,500 g (aOR: 1.15, 95% CI [1.05, 1.26]), and HDP (aOR: 1.27, 95% CI [1.09, 1.36]).

**Table 2:** Relationship between dietary inflammatory index and obstetrics outcomes

	Quartiles of DDI			
	Q1 (*n* = 22,420)	Q2 (*n* = 22,350)	Q3 (*n* = 22,349)	Q4 (*n* = 22,255)
	Most antiinflammatory			Most proinflammatory
PTB < 37 weeks				
OR (95% CI)	1 (Ref)	0.98 (0.90–1.07)	1.03 (0.95–1.12)	0.99 (0.91–1.08)
aOR (95% CI)	1 (Ref)	0.99 (0.91–1.08)	1.05 (0.97–1.15)	1.02 (0.94–1.11)
PTB < 34 weeks				
OR (95% CI)	1 (Ref)	1.02 (0.84–1.23)	1.08 (0.90–1.30)	1.22 (1.01–1.46)
aOR (95% CI)	1 (Ref)	1.04 (0.86–1.25)	1.12 (0.93–1.35)	1.29 (1.07–1.55)
LBW < 2,500 g				
OR (95% CI)	1 (Ref)	0.98 (0.92–1.05)	1.05 (0.98–1.12)	1.07 (1.00–1.15)
aOR (95% CI)	1 (Ref)	0.98 (0.92–1.05)	1.06 (0.99–1.13)	1.08 (1.01–1.16)
LBW < 1,500 g				
OR (95% CI)	1 (Ref)	0.89 (0.69–1.14)	1.09 (0.86–1.38)	1.07 (0.84–1.36)
aOR (95% CI)	1 (Ref)	0.91 (0.71–1.17)	1.14 (0.89–1.44)	1.15 (0.90–1.47)
SGA				
OR (95% CI)	1 (Ref)	0.92 (0.85–1.00)	0.94 (0.86–1.02)	1.01 (0.93–1.10)
aOR (95% CI)	1 (Ref)	0.92 (0.84–1.00)	0.93 (0.86–1.01)	0.99 (0.91–1.10)
HDP				
OR (95% CI)	1 (Ref)	1.01 (0.90–1.13)	1.02 (0.91–1.14)	1.15 (1.03–1.28)
aOR (95% CI)	1 (Ref)	1.04 (0.93–1.17)	1.08 (0.96–1.21)	1.27 (1.09–1.36)

*Note*. aOR was calculated by logistic regression analysis, using maternal age, body mass index before pregnancy, maternal smoking status, maternal education, and household income.

Abbreviations: aOR, adjusted odds ratio; CI, confidence interval; DII, dietary inflammatory index; HDP, hypertensive disorder of pregnancy; LBW, low birthweight; OR, odds ratio; PTB, preterm birth; Ref, reference; SGA, small for gestational age.

## 10 DISCUSSION

This is the first study to investigate the relationship between inflammatory diet and obstetric outcomes, using the largest birth cohort study in Japan. Our results suggest that proinflammatory diet before pregnancy was a risk factor for PTB < 34 weeks and LBW <2,500 g, and HDP.

Until now, few studies that examined the correlation between DII score during pregnancy and obstetrics outcomes have conducted. Sen et al. (2016) calculated the DII score during pregnancy using 28 dietary parameters and examined the association between the quartile of DII score and obstetric outcomes in 1,808 maternal participants. They reported that a proinflammatory diet during pregnancy was associated with maternal systematic inflammation and may be associated with fetal growth restriction. Our result that proinflammatory diet before pregnancy is related to maternal systematic inflammation in term of leukocytosis (defined WBCs > 12,000) is consistent with the findings of that study. Contrary to that study, our study indicates that proinflammatory diet before pregnancy is associated with PTB < 34 weeks, LBW < 2,500 g, and HDP but not with growth restriction. We assumed that the reasons for these discrepancies are the differences in sample size, definition of obstetrics outcome, assessment time of diet content (during or before pregnancy), and statistical methods for calculating the risk of obstetrics outcomes. In the present analysis, we applied quartiles of DII as dummy variable, leaving the Q1 group (the most antiinflammatory diet) as reference to calculate the aOR of each DII category (Q2, Q3, and Q4) for obstetrics outcomes in the logistic regression model. We thought that this logistic model enabled the evaluation of the risk of adverse obstetrics outcomes in every category of participants.

The mechanism of the association between proinflammatory diet before pregnancy and PTB is unknown. We think that there are two scenarios where proinflammatory diets cause PTB. First, some foods with large amounts of saturated and transfats induce proinflammatory cytokines (Soto‐Vaca, Losso, McDonough, & Finley, 2013). Proinflammatory cytokines, such as IL and TNF, produce prostaglandin and matrix‐degrading enzymes. Prostaglandin stimulates uterine contractions, whereas degradation of the extra cellular matrix leads to preterm rupture of fetal membranes, resulting in spontaneous PTB. In animal studies, Manuel, Latuga, Ashby, and Reznik (2019) reported that a high‐fat diet leads to gut dysbiosis and dysregulated uterine expression in pregnant mice, resulting in PTB. They also reported immune tolerance induced by endotoxin priming prevents high‐fat diet dams from PTB (Manuel et al., 2019). Other studies reported that alterations in the composition and function of gut microbiota, such as dysbiosis, contribute to systematic inflammation (Laugerette, Vors, Peretti, & Michalski, 2011). Therefore, it might be reasonable to assume that systematic inflammation as a significant increase in WBCs in the proinflammatory diet group was related to PTB. However, PTB has the same endpoint, consisting of two clinical subtypes: spontaneous PTB and medically indicated PTB, which is conducted for cases of SGA or HDP (Kyozuka et al., 2018). Habitual proinflammatory diet has associations with regulation of inflammation, leading to modulation of the atherogenesis process and endothelial dysfunction (Barbaresko, Koch, Schulze, & Nöthlings, 2013). The atherogenesis process and endothelial dysfunction play a major role in the pathogenesis of hypertensive disorder (Ramallal et al., 2015; Roberts & Redman, 1993). Therefore, we speculate another scenario for the increase in PTB < 34 weeks among the proinflammatory group; this might be due to an increase in medically indicated PTB, such as HDP (a cardiovascular event induced by proinflammatory diet habits).

The strength of this study is that it is the first large‐scale study conducted in Japan by the Japanese government with meticulous attention to data collection. Therefore, this study is considered to be representative of the general pregnant population in Japan (Yamaguchi et al., 2018). Nevertheless, this study also has potential limitations to be considered. First, although we measured the WBC during first trimester, we did not measure or include data on plasma inflammatory cytokines, such as CRP, IL‐6, TNFα, or another inflammation marker in the present study. These data could augment the correlation between proinflammatory diet and PTB. Second, although we accounted for some confounding factors in large portions of the questionnaire, unknown factors which may affect the occurrence of PTB, LBW, or HDP might have existed. Third, the DII score of each participant in the present study was calculated using only the FFQ of JECS participants who were Japanese women and not validated to other ethnics yet. Therefore, our results may not be applicable to other ethnicities. Fourth, because the FFQ used for calculating the DII score in the present study was based on self‐reported information during their first trimester, recall bias may be possible as participants might have had morning sickness and were asked to recall their diet content before pregnancy.

In conclusion, we found that a proinflammatory diet before pregnancy was a risk factor for PTB, LBW, and HDP. Our study suggests that dietary habits may affect obstetric outcomes. Therefore, proinflammatory diet needs to be controlled to improve perinatal prognosis.

## SOURCES OF FUNDING

None.

## CONFLICTS OF INTEREST

The authors declare that they have no conflicts of interest.

## CONTRIBUTIONS

All authors approved the final manuscript. M. I. initiated the concept and designed the study to which H. K., A. Y., K. F., and K. H. gave advice. M. K., A. S., and Y. O. collected the data. M. I. analysed the data and wrote the manuscript. M. H., K. F., S. Y., M. K., A. S., Y. O., K. H., and the JECS group reviewed the manuscript and gave critical advice.
